# The Medical Research Committee

**Published:** 1913-06-28

**Authors:** 


### The Hospital

The Workers' Newspaper of Administrative Medicine and Institutional Life, Administration, National Insurance and Health.

No. 1409, Vol. LIV. SATURDAY, JUNE 28, 1913. PRICE ONE PENNY.

### THE MEDICAL RESEARCH COMMITTEE.

Whatever be the controversial passions of the hour, medical practitioners generally will welcome the appointment, recently announced, of a Medical Research Committee in connection with the administration of the National Insurance Act. The composition of the Committee, too, will, we believe, command the confidence of the profession, and it is, in our judgment, all to the good that such a Committee should include members whose point of view is not that of the mere expert. Medical research is a term of which much has been heard in recent years, and it has, we think, very frequently received an unduly narrow interpretation. To many minds, even yet, it carries the suggestion of something done or attempted in a laboratory—this and nothing else. It means chemical, bacteriological, or microscopical investigations; these and these alone, it is assumed, can claim the countenance and form of a truly scientific inquiry. No one will for a moment question the value and indeed the necessity for such activities, But laboratory methods and results do not and cannot touch at all points the complex problems which are associated with the cause and prevention of disease. Confident, clear cut, and precise in form as these results often appear, they deal almost necessarily with narrow and limited issues, and they require to be set in an appropriate perspective. The natural enthusiasm of the mere laboratory worker, and the definiteness of his observations, are apt to excite an unduly dogmatic confidence and a disposition to refuse a hearing to all observations and proposals which cannot boast the sanction of so-called scientific reactions and tests. As bearing on the same issue, also, it has to be remembered that the conditions of laboratory experiment are by no means identical with those which surround the processes of disease as these display themselves in the theatre of human life. What in the very nature of things is more or less artificial cannot be applied to the natural sphere without some measure of hesitation and qualification. In short, while, in any scheme of comprehensive medical research, laboratory investigations must needs hold a considerable and important place, they must be checked and supplemented by other inquiries. We must hear the voices of those who have opportunities for studying the problems of public health from a broad and human standpoint; we must summon to our aid the scientific statistician; and last, but by no means least, we must invoke the assistance of those who, whether at the bedside or in the *post-mortem* theatre, are daily in contact with the exhibitions of disease in individual men and women. It is only by the union and co-ordination of knowledge from these various quarters that a solid body of sound information capable of carrying general doctrines and practical measures can be secured.

There is every reason to believe that the new Medical Research Committee will fully recognise the obligation which falls upon them to welcome truths from whatever quarter these may arrive. To anticipate some prompt and dramatic results from the appearance of the Committee would be to shut one's eyes to the lessons of history. The immediate task is the collection of large bodies of well-established facts; and next, to inspire and direct investigations where experience shows that the knowledge necessary to the solution of the complete problem is defective. All this will be a laborious process; it will be also a slow process. That there will be pressure towards haste is almost certain. The public generally has a somewhat pathetic faith in committees of distinguished men, and it pines for definite conclusions. It would not be difficult to find some ground for believing that committees owning an official paternity are liable to pressure from the powers which have called them into existence. The natural desire is to do something, and to do it quickly. Hence on not a few occasions hurried conclusions have led to premature action, with waste of money, of time and of effort, and bitter disappointment, as natural consequences. History in these respects, it is to be earnestly hoped, will not repeat itself on the present occasion. But if this is to be avoided, there will be need for the Committee to show a resolute front alike to public opinion and to official pressure. Perhaps the most obvious illustration of the need for secure and cautious movement is to be found in the problem of tuberculosis. This involves large economic questions and, more than possibly, a large public expenditure. Will anyone say that there exists at the present moment so definite and well-established a body of fact in relation to the treatment of tuberculosis as to secure that such expenditure shall be wisely directed? And if this is true of a disease frequent in occurrence, widely distributed, and carefully studied, how much more forcible is the appeal in relation to less well-known and less common diseases. The prevention and treatment of tuberculosis stands in the forefront as a problem demanding the most exact and impartial study. We are hopeful that neither in this nor in any other direction will the new Committee allow itself to be driven into either hurried conclusions or premature action.

